# Unlocking intergenerational support: The impact of long-term care insurance on elderly family dynamics

**DOI:** 10.1371/journal.pone.0337073

**Published:** 2025-11-21

**Authors:** Yi Qu, Yumeng Li, Anning Yin, Jia Li

**Affiliations:** 1 School of Public Administration, Dongbei University of Finance & Economics, Dalian, China; 2 School of Public Administration, Dongbei University of Finance & Economics, Dalian, China; 3 School of Public Administration, Dongbei University of Finance & Economics, Dalian, China; 4 School of Public Administration, Dongbei University of Finance & Economics, Dalian, China; University of Saskatchewan, CANADA

## Abstract

As China faces an aging population and changing family dynamics, including smaller nuclear families and increased mobility, it is crucial to understand how long-term care insurance policies affect intergenerational support for older adults. This study investigates the impact of these policies on economic, caregiving, and emotional support for individuals aged 60 and above. Using multi-period difference-in-differences models and unbalanced panel data from the CHARLS database (2011–2020), the analysis focuses on 66 Chinese pilot cities implementing long-term care insurance policies, emphasizing urban households and healthier elderly groups. The findings show that long-term care insurance policies significantly reduce children’s financial and caregiving burdens while strengthening emotional bonds between older parents and their children. These effects are stronger in urban areas and among healthier elderly populations, indicating that household context and health status influence policy benefits. The results highlight the role of these policies in easing intergenerational pressures amid demographic shifts and provide evidence to improve policy design. By revealing differences in policy effectiveness across family types, this study offers theoretical insights for developing targeted eldercare strategies. It supports government efforts to tackle aging-related challenges and promote sustainable family support systems. The research underscores the need for flexible policies that respond to evolving societal needs and ensure equitable care for China’s elderly.

## 1. Introduction

Reciprocal support and care among family members are essential for the physical and mental well-being of the elderly [1]. As the role of families in providing daily care and promoting healthy aging becomes increasingly important, they also face growing pressures [2]. On one hand, the aging population is intensifying, leading to a significant national and societal burden related to eldercare [3]. On the other hand, changing fertility norms have resulted in smaller family sizes and declining birth rates, which reduce internal resources for elder support and disrupt intergenerational assistance [4]. Moreover, the proportion of disabled elderly individuals is rising, while the development of nursing homes and services has not kept pace with demand [5], exacerbating shortages in long-term care resources. Despite these challenges, existing research on the diminishing role of families in caregiving--combined with policy biases--has resulted in underdeveloped theoretical frameworks concerning familial caregiving roles [6].This gap highlights the need to address how to enhance the quality of life for older adults while alleviating the burdens of familial caregiving. Resolving this issue has become increasingly urgent.

Data from the China Center for Disease Control and Prevention (2021) reveal that 16% of elderly individuals experience partial or complete disabilities, with 4.8% classified as fully disabled. Meanwhile, China’s integrated medical-nursing service system remains underdeveloped compared to other advanced nations, highlighting an urgent need for improvement [7]. Against this background of escalating care needs and infrastructural gaps, policymakers have prioritized the expansion of social safety nets [8]. Currently, a pilot long-term care insurance scheme has been implemented across 49 cities, and preliminary findings suggest that this insurance positively affects both the quality of life and caregiving services for older adults [9].

However, there is no consensus on whether long-term care insurance serves as a “crowding out” or “crowding in” mechanism for emotional support within families [10]. Disparities arising from regional development differences between urban and rural areas, as well as variations in family structures [11], contribute to significant variability in the intergenerational support received by elderly individuals from their children [12]. Additionally, while research on the effects of social security systems on intergenerational support has increased [13], it predominantly focuses on public transfer payment models.

This study is grounded in the theory of altruism, highlighting the voluntary distribution of resources among family members motivated by concern for others and a desire to help [14]. In the context of intergenerational support, children’s supportive behaviors are viewed as expressions of care for their parents, rather than as attempts to gain personal benefits [15]. This emotional bond may prompt children to adjust their internal support patterns when faced with external resources, such as long-term care insurance, which can affect the overall allocation of resources within the family. Thus, this research addresses the following primary question:

### 1.1. What is the impact of long-term care insurance on intergenerational support in families caring for older adults?

This study offers original insights into the existing literature across five key dimensions. First, it uses long-term care insurance as a focal point to explore its effects on intergenerational support, thereby enriching the theoretical framework surrounding this topic. Second, by analyzing how long-term care insurance policies impact intergenerational support across various family types, income levels, and health statuses among older adults, this research clarifies the potential policy roles in reallocating internal resources and shaping caregiving behaviors. Third, it examines how long-term care insurance policies influence the relationships between formal and informal caregiving arrangements, while also exploring how these policies prompt adjustments in familial dynamics. Fourth, the study underscores the capacity of long-term care insurance to alleviate burdens related to family caregiving and improve the quality of life for older adults, providing empirical evidence that can inform government evaluations and adjustments to such policies. Finally, through quantitative analyses of how long-term care insurance systems affect intergenerational support mechanisms, this research investigates preferences for different forms of elderly caregiving and their variations, offering empirical support for differentiated allocations within social elderly care services.

The remainder of the paper is structured as follows: Section 2 presents a literature review, Section 3 outlines the methodology applied, and Section 4 analyzes the results. Section 5 provides a comprehensive discussion of the research findings, while Section 6 delineates the limitations of the study and outlines potential directions for future research.

## 2. Literature review

To develop a thorough understanding of the current research landscape and identify knowledge gaps, we reviewed relevant literature related to our topic. Below, we outline three key research streams that are particularly significant to our study.

### 2.1. The concept and influencing factors of intergenerational support for the elderly

Intergenerational support within families encompasses the emotional and material assistance exchanged among family members, particularly between the elderly and their children [16]. Previous research shows that this support significantly impacts the psychological health and life satisfaction of older adults [17]. Emerging evidence further underscores that a robust family support system, which complements the social security mechanism, can effectively reduce physical health risks and narrow health disparities among the elderly [18]. It can take the form of financial assistance, emotional care, and practical help, all aimed at enhancing the social connections and quality of life for the elderly [19,20].

In terms of economic support, extant studies indicate that when older adults receive more social benefits, financial assistance from their children may decrease, a phenomenon known as the “crowding out effect” [21]. Conversely, other research suggests that external social security systems can enhance children’s support for their parents, leading to a “crowding in effect” [22].

For older adults, especially those in need of care, fulfilling individual social needs is essential for realizing personal value and engaging with society.[23,24]. Support from children can effectively reduce feelings of loneliness among the elderly [25]. Additionally, factors such as age, gender, educational level, and living environment play a role in influencing the life satisfaction of older adults [26]. Spousal caregivers often experience significant mental stress due to declining health and caregiving responsibilities [27], highlighting the critical importance of intergenerational support.

### 2.2. The impact of social security on intergenerational support

Social security refers to protective measures provided by the government to enhance individual quality of life [28]. Existing research indicates that social security has a significant impact on intergenerational support [29]. On one hand, social security interventions can elevate the living standards of older adults, alleviating the pressure on family caregiving [18]. Studies have shown that improvements in social security may lead to reduced financial support from children to their parents, resulting in a “crowding out effect” [30]. On the other hand, enhanced social security systems can promote mutual assistance among family members, creating a “crowding in effect” [31]. A robust social security system not only eases the economic burden on the elderly but also strengthens supportive relationships within families [18,32]. Specifically, social security can facilitate communication and interaction between the elderly and their children, thereby enhancing family cohesion. Moreover, intergenerational support, particularly in the form of financial assistance and regular communication from children who do not cohabit with their elderly parents, has been found to substantially enhance the life satisfaction of the elderly [33].

In many cases, economic support can translate into emotional support [34], increasing life satisfaction for older adults. Conversely, a lack of effective social security may weaken intergenerational support relationships, leading to increased feelings of loneliness and depression among the elderly [35].

In China, as the social security system gradually improves, patterns of intergenerational support are evolving. The traditional family care model, which emphasizes the belief in “raising children for old age,” is transitioning toward a model that relies more on social security [36,37]. This shift may influence the supportive behaviors among family members.

### 2.3. The relationship between long-term care insurance and intergenerational support

Long-Term Care Insurance (LTCI) is a formal arrangement that provides financial compensation and support services for individuals who require long-term care due to physical decline, chronic illness or disability. Its primary objective is to alleviate the financial strain and caregiving burden on families through a risk-sharing mechanism [1]. Older adults who have long-term care requirements constitute a risk factor for poverty and socioeconomic deprivation within their households, Such households often face multiple pressures, including having insufficient purchasing power to pay for care services, a decline in the labor participation rate among family members, and healthcare expenditure taking precedence over basic living expenses [38,39]. In terms of institutional effectiveness, LTCI alleviates the burden on family caregivers by providing standardized care services for the elderly, such as home-based care, day care and subsidies for institutional care, alongside targeted financial support. Specifically, the implementation of long-term care insurance typically increases the likelihood that older adults will opt for formal caregiving [40], thereby reducing reliance on informal family care. This effect has been widely recognized in the experience of other countries. Since Japan introduced its Long-Term Care Insurance (LTCI) scheme in 2000, the use of formal care services has increased significantly, particularly among elderly people living alone and those with mild disabilities. At the same time, the proportion of people relying solely on informal family care has declined [41]. This phenomenon not only reflects a shift in caregiving responsibilities but may also affect the relationships and support dynamics among family members [42].

Research has identified that long-term care insurance can enhance the quality of intergenerational support [28], fostering mutual understanding and assistance between older adults and their children. Such insurance not only helps families cope with caregiving costs but also strengthens emotional bonds among family members, thereby improving overall life satisfaction [43]. Long-term care for the elderly in need of care is a key issue affecting the quality of life of family caregivers. The long-term burden of care will aggravate the negative emotions of family caregivers, such as depression [44]. Existing studies have already focused on the positive impact of Long-Term Care Insurance (LTCI) on the quality of intergenerational support. By providing appropriate caregiving services, long-term care insurance helps alleviate the psychological stress of family caregivers [45], further enhancing the effectiveness of intergenerational support. Long-Term Care Insurance (LTCI) serves as a protective mechanism for the policyholder and as a ‘family liberation mechanism’. It significantly alters intergenerational care expectations and behavioral patterns, freeing family members, particularly adult children, from the potential burden of caregiving responsibilities. This gives them greater freedom to arrange their personal lives and career development, enter the labor market and increase their household’s economic capacity [46]. Overall, long-term care insurance effectively supports the caregiving needs of families and promotes the quality of life for older adults [47].

### 2.4. Summary

Intergenerational support within families is crucial for enhancing the quality of life for older adults, especially in the face of health and economic challenges [48]. With the development of social security systems, particularly the implementation of long-term care insurance, the patterns of intergenerational support are experiencing significant changes [49,50].

Existing research has yet to provide a unified theoretical explanation for the effects of long-term care insurance (LTCI), with two competing perspectives prevailing. Namely, the introduction of LTCI may generate both “crowding-in” and “crowding-out” effects. Altruism suggests that the support behaviors exhibited by family members across generations stem from a concern for the well-being of older adults, and that these behaviors become more important as the well-being of older adults improves [51]. From an altruistic perspective, family members provide support until the marginal utility is equal to the marginal cost. The introduction of long-term care insurance (LTCI) reduces the marginal utility of children providing additional support by directly improving the welfare of the elderly. This creates a “substitution effect” on informal family support, whereby government-provided services directly replace the care contributions originally made by family members. Consequently, informal support among family members diminishes, leading to the “crowding out” of intergenerational support [52]. Exchange theory offers an alternative explanation, positing that intergenerational support is an implicit, reciprocal exchange. This exchange can be immediate, such as money in exchange for care, or intertemporal, such as present care in exchange for future inheritance [53]. In the context of long-term care insurance (LTCI), exchange theory predicts the potential emergence of a “crowding-in” effect. By alleviating the burden of physical caregiving, LTCI enables adult children to provide emotional companionship and financial support at a lower cost, thereby increasing the supply of such support [54].

Thus, the impact of long-term care insurance on intergenerational support is complex and warrants further investigation. Accordingly, we propose the following hypothesis:

**H1:** Long-term care insurance significantly influences intergenerational support, although the direction of this influence remains uncertain.

With long-term care insurance providing formal economic security, family members--especially children--may reduce their financial support for older adults, resulting in a “crowding out” effect. This effect suggests that children may perceive their financial responsibilities as diminished due to the presence of insurance, thereby weakening the economic support network within the family. Furthermore, a decrease in economic support may lead to reduced dependency of older adults on their families. Therefore, we propose:

**H2:** Long-term care insurance reduces intergenerational economic support.

Long-term care insurance not only provides economic security but may also enhance the emotional connections between children and older adults. By alleviating caregiving pressures, children can better engage in emotional support, increasing the frequency of family interactions and improving the psychological well-being and life satisfaction of older adults. Effective emotional support can enhance family cohesion and promote positive intergenerational relationships. Therefore, we propose:

**H3:** Long-term care insurance increases intergenerational emotional support.

In terms of caregiving needs, families of disabled elderly individuals may have a higher reliance on long-term care insurance; the intervention of such insurance could significantly alleviate caregiving burdens and enhance the supportive relationships among family members. In contrast, healthier older adults may be more actively involved in social activities and family interactions, further improving the quality of intergenerational support. Hence, long-term care insurance may exert different effects across these two-family types. Therefore, we propose:

**H4:** The intergenerational behaviors of families with disabled elderly individuals/healthy elderly individuals are more significantly influenced by long-term care insurance.

The frequency of hospitalizations reflects the health status and caregiving needs of older adults. The implementation of long-term care insurance can effectively reduce hospitalization rates, thereby alleviating the pressures on family caregivers. This mediating effect aids in understanding how long-term care insurance impacts intergenerational support by improving the health status and caregiving needs of older adults. A reduction in hospitalization not only lessens the economic burden on families but also increases interactions among family members, thereby enhancing emotional support. Thus, we propose:

**H5:** The frequency of hospitalizations serves as a mediating variable in the impact of the long-term care insurance system on the daily caregiving of older adults.

## 3. Materials and methods

### 3.1. Database selection

The data used in this study is derived from the China Health and Retirement Longitudinal Study (CHARLS). CHARLS is a large-scale interdisciplinary research project that encompasses a wide range of information regarding the basic characteristics of the elderly, family structures, economic support, and health status. During the data collection process, CHARLS employed a random sampling method, covering 450 communities across the nation, with a total sample of 19,000 respondents from 12,400 households, ensuring a high level of representativeness.

The CHARLS database offers multiple advantages. Firstly, long-term care insurance is aimed at middle-aged and elderly individuals with potential disability risks or those who are already disabled and in need of care. Its impact on the social pension system is most evident among the elderly population. The respondents of CHARLS consist of urban and rural residents aged 45 and above, aligning with the coverage of our study’s target population. Secondly, this paper explores the factors related to intergenerational support within the context of long-term care, controlling for individual and family-level variables. CHARLS provides comprehensive data across four dimensions: individual, family, community, and macro policy, offering robust empirical support for this research. Finally, the CHARLS database includes differentiated data coding for various provinces and municipalities. This study uses a multi-period difference-in-difference (DID) model to evaluate the effects of the long-term care insurance pilot policy. To enhance transparency, we clarify the selection criteria for the pilot and control groups. The experimental group comprises cities designated as long-term care insurance pilot cities by the National Health Security Administration between 2011 and 2018. The control group consists of cities that did not implement long-term care insurance policies during the same period.

The five waves of data collected in 2011, 2013, 2015, 2018, and 2020 cover the pilot period from 2012 to 2020, aligning with the requirements of this study and fulfilling the conditions for a multi-time-point difference-in-differences model.

This study used publicly available, de-identified data from the China Health and Retirement Longitudinal Study (CHARLS). The research was reviewed and approved by the Ethics Committee of the School of Public Administration, Dongbei University of Finance and Economics (Approval waived due to use of anonymized secondary data). All procedures were conducted in accordance with the Declaration of Helsinki.

### 3.2. Variable setting

In this study, the explained variable is the intergenerational support provided by children to their parents. Based on the CHARLS questionnaire, this explained variable is categorized into three dimensions: economic support, emotional support, and daily care. Economic support primarily consists of the cash transfers made by children to their parents, emotional support is measured by the frequency of visits between children and parents, and daily care is assessed based on whether children care for their parents and the amount of time dedicated to such care.

The core explanatory variable in this study is whether the surveyed household belongs to a city that is part of the long-term care insurance pilot program and whether they benefit from it. After filtering the data, the qualifying samples are designated as the experimental group and assigned a value of 1; otherwise, they are assigned a value of 0.

The control variables in this study are categorized into three dimensions: individual characteristics, socioeconomic variables, and family characteristics. Individual characteristics include age, gender, education level, marital status, chronic illness status, disability status, and self-reported health status. Socioeconomic variables encompass pension type and household registration type, while family characteristics include the number of children and total household income. All variables are listed in Table 1.

**Table 1:** Description of key variables.

Key Variables		Variable Description	Assignment Rules
Dependent Variable	Intergenerational support from children to parents	Total cash transfer payments from children to parents (Economic Support)	Total Cash Support
		Frequency of visits from children to parents (Emotional Support)	Frequency assigned values from 0 to 8
		Care provided by children in daily life (Daily Care)	Care Situation and Duration
Independent Variable	Long-term care insurance	Whether the surveyed household is in a pilot city for long-term care insurance	Condition met = 1
			Condition not met = 0
Control Variables	Individual characteristics	Age of elderly individuals (60 years and older)	Direct Age Value
		Gender of elderly individuals	Male = 1
			Female = 0
		Educational attainment of elderly individuals	Educational Attainment assigned values from 0 to 19
		Marital status of elderly individuals	Married = 1
			Unmarried = 0
		Whether the individual has at least one chronic disease	Has a disease = 1
			No disease = 0
		Whether there is any ADL disability	Disabled = 1
			Not disabled = 0
		Self-rated health status	Assigned values from 1 to 5
	Socioeconomic factors	Whether the individual participates in pension insurance	Insured = 1
			Not insured = 0
		Household registration type	Rural = 1
			Non-rural = 0
	Family characteristics	Number of children	Includes biological and adopted children
		Total household income	Sum of Transfer Payments

After reviewing, filtering, and processing the data, we obtained a total of 35,971 sample data points from 125 cities, resulting in an unbalanced panel dataset. Among these, there are 28 cities in the experimental group and 97 cities in the control group. The experimental group comprises 945 samples, while the control group includes 35,062 samples. Table 2 presents an overview of the dependent variables, independent variables, and control variables within the elderly sample.

**Table 2:** Overview of variables in the elderly sample.

Variables		Total Sample			
		Mean	Standard Deviation	Min	Max
Dependent Variables	Economic Support	3,217	5,835	0	36,000
	Frequency of Visits	6.09	4.06	0	39
	Daily Care	105.8	246.5	1	4,764
Control Variables	Age	68.6	6.81	60	87
	Gender	0.49	0.50	0	1
	Educational Attainment	4.46	5.37	0	16
	Marital Status	0.78	0.42	0	1
	Chronic Illness	0.72	0.45	0	1
	Disability Status	0.32	0.47	0	1
	Number of Children	3.12	1.48	0	7
	Pension Insurance	0.83	0.37	0	1
	Household Registration Type	0.75	0.43	0	1
	Self-Rated Health	3.07	0.98	1	5
	Total Household Income	27,520	257,472	0	3.906e + 07
	Observations	35971	35971	35971	35971

Table 3 presents the descriptive statistics resulting from the division of the overall sample into experimental and control groups. The descriptive statistics provide a clear illustration of the changes and differences in the intergenerational economic support, emotional support, and daily care received by elderly parents in pilot cities versus non-pilot cities from 2011 to 2018.

**Table 3:** Descriptive statistics of the sample by experimental and control groups.

Variables		Non-pilot cities		Pilot cities		T-test results	
		Mean	Standard Deviation	Mean	Standard deviation	T value	P value
Dependent Variables	Economic Support	3016	5757	2714	6197	−6.48	0.000
	Frequency of Visits	1306	2277	2307	3144	−14.34	0.000
	Daily Care	106.6	248.0	65.59	158.6	0.6791	0.4973
Control Variables	Age	68.63	6.81	68.85	6.90	−1.04	0.2980
	Gender	0.49	0.50	0.52	0.50	−1.74	0.0814
	Educational Attainment	4.44	5.38	5.57	4.64	−6.97	0.000
	Marital Status	0.78	0.42	0.84	0.37	−4.65	0.000
	Chronic Illness	0.73	0.45	0.63	0.48	−6.97	0.000
	Disability Status	0.33	0.47	0.22	0.41	7.36	0.000
	Number of Children	3.14	1.48	2.20	1.18	21.16	0.000
	Pension Insurance	0.83	0.38	0.93	0.25	−8.97	0.000
	Household Registration Type	0.76	0.43	0.47	0.50	22.49	0.000
	Self-Rated Health	3.08	0.98	2.82	1.02	8.17	0.000
	Total Household Income	35062	35062	945	945	35971	35971
	Observations	3016	5757	2714	6197	−6.48	0.000

### 3.3. Model selection

This study employs the difference-in-differences (DID) method to identify the impact of long-term care insurance on intergenerational support within families. The baseline linear regression model is specified as follows:


$$Y_{hct}=\alpha +\beta {treat}_{c}*{time}_{t}+\delta X_{hct}+\tau_{t}+\omega_{h}+\varepsilon_{ct}$$

In this model, the subscripts *h, c,* and *t* represent household, city, and time, respectively. The model includes a set of dependent variables that reflect the intergenerational support received by the elderly parents, which encompasses the amount of cash support received, the value of in-kind support, the frequency of visits by children, and the frequency of communication with children. The core explanatory variable is the interaction term between the treatment group dummy variable and the dummy variable indicating the period before and after the implementation of long-term care insurance in pilot cities, which denotes the status of the long-term care insurance trial.

Specifically, the treatment group dummy variable indicates whether the surveyed household is in a pilot city for the long-term care insurance system, assigning a value of 1 if it is and 0 otherwise. The policy trial time dummy variable is set to 1 for the years following the implementation of the policy and 0 for the years prior. The estimated coefficient reflects the average treatment effect of long-term care insurance on the intergenerational support received by elderly parents. Additionally, the model includes a set of control variables at both the individual and parental levels, as well as fixed effects for year and household, along with a random disturbance term.

## 4. Results

Based on the multi-period DID differential model established above, this study uses the processed CHARLS non-equilibrium panel data in 2011, 2013, 2015, 2018 and 2020 for regression analysis. To evaluate the effects of long-term care insurance policies on intergenerational financial support, emotional support and daily care for the elderly. To ensure the accuracy of the analysis, all explained variables have been treated logarithmically. The regression results are shown in Tables 4–.

**Table 4:** Baseline regression results of long-term care insurance on intergenerational economic support for the elderly.

Variable	Intergenerational Economic Support
Time*treat	−0.310***
	(0.119)
Age	−0.023
	(0.026)
Sex	0.089
	(0.251)
Educational level	−0.004
	(0.006)
Marital status	0.161***
	(0.051)
Chronic disease	0.122***
	(0.030)
Disability condition	0.013
	(0.024)
Self-rated health	0.009
	(0.013)
Endowment insurance	0.010
	(0.029)
Type of household registration	−0.020
	(0.068)
Number of children	0.045*
	(0.025)
Constant term	8.378***
	(1.678)
r2	0.062

Standard error in brackets: ***p < 0.01,**p < 0.05,*p < 0.1

**Table 5:** Baseline regression results of long-term care insurance on intergenerational emotional support for the elderly.

Variable	Intergenerational Economic Support
Time*treat	0.052**
	(0.026)
Age	−0.007
	(0.008)
Sex	0.052
	(0.064)
Educational level	−0.003*
	(0.002)
Marital status	0.045***
	(0.015)
Chronic disease	0.008
	(0.009)
Disability condition	−0.007
	(0.007)
Self-rated health	0.002
	(0.004)
Endowment insurance	0.004
	(0.009)
Type of household registration	−0.008
	(0.021)
Number of children	0.180***
	(0.007)
Constant term	1.521***
	(0.498)
r2	0.056

Standard error in brackets: ***p < 0.01,**p < 0.05,*p < 0.1

**Table 6:** Results of long-term care insurance on intergenerational daily care of the elderly.

Variable	Intergenerational Economic Support
Time*treat	−0.036*
	(0.019)
Age	−0.002
	(0.008)
Sex	0.010
	(0.027)
Educational level	−0.018
	(0.015)
Marital status	−0.020
	(0.032)
Chronic disease	0.005
	(0.014)
Disability condition	0.084***
	(0.014)
Self-rated health	0.013*
	(0.007)
Endowment insurance	−0.018
	(0.015)
Type of household registration	−0.012
	(0.037)
Number of children	−0.017
	(0.012)
Constant term	0.384
	(0.519)
r2	0.016

Standard error in brackets: ***p < 0.01,**p < 0.05,*p < 0.1

### 4.1. The impact of long-term care insurance on intergenerational financial support for the elderly

The results indicate that long-term care insurance policies significantly impact the intergenerational financial support provided to the elderly. As shown in Tables 4–6, the implementation of long-term care insurance reduced the financial support received by older adults by 31%, with this finding being significant at the 1% level. One possible explanation is that long-term care insurance lowers the cost of care for the elderly and enhances their financial security, thereby reducing their dependence on financial support from their children.

Additionally, the regression results reveal that individual characteristics, particularly marital status and the presence of chronic diseases, significantly affect older adults’ access to intergenerational economic support. Specifically, older adults with spouses receive higher financial support compared to those without spouses. Moreover, older individuals with chronic diseases tend to receive more financial assistance from their children; however, disability—another health condition—was not found to be significant.

### 4.2. The impact of long-term care insurance on intergenerational emotional support for the elderly

In terms of intergenerational emotional support, the regression results showed that the coefficient direction of the explained variable treat*time was positive, indicating that long-term care insurance enhanced the emotional connection between children and parents to a certain extent compared with non-pilot areas.

Specifically, participation in long-term care insurance resulted in a 5.2% increase in parent-child visits, which was significant at the 5% significance level. Several factors within China’s institutional culture may explain this phenomenon. First, long-term care insurance indirectly alleviates the caregiving burden on children by assuming part of the financial responsibility. Second, East Asian family ethics may create social space for children to express emotional care under reduced economic pressure, making emotional support a more prominent dimension of caregiving. Thus, long-term care insurance strengthens their willingness to care for their parents, thereby reinforcing family bonds.

### 4.3. Influence of long-term care insurance on intergenerational care of the elderly

The implementation of long-term care insurance also has a crowding out effect on daily care time. According to Tables 4, 5, after enrolling in long-term care insurance, children’s daily care time for their parents decreased by 3.6%, which is significant at the 10% level. This decline may stem from multiple factors. From a substitution effect perspective, the payment structure of long-term care insurance may be the cause. Clear cost-reimbursement mechanisms lower the financial barrier for the elderly, making professional care more accessible and increasing their preference for standardized, professional, formal care services. This shift in preference naturally reduces their reliance on non-professional care provided by children when socialized services covered by insurance can meet their needs. Consequently, there is a decrease in the amount of time children spend caregiving. Labor migration may also be a significant underlying factor that contributes to reduced caregiving time among adult children. Long-term care insurance can alleviate some of the constraints on labor mobility imposed by caregiving responsibilities. When parents have access to socialized care, adult children who work away from home are less likely to frequently return home to fulfill caregiving duties or abandon job opportunities elsewhere due to care needs. This indirectly reduces the time they actually spend providing care.

### 4.4. Robustness test

When regression analysis is performed, the random disturbance term will affect the explanatory variables, causing the data to violate the Gauss-Markov theorem, resulting in a deviation in parameter estimation, which is called the endogeneity problem. In the differential model (DID) for analyzing policy effects, many data are non-experimental, which makes the endogeneity problem appear in a large amount of data. This is mainly due to the improper setting of the measurement model, such as variable bias, sample selection bias, simultaneous bias, measurement bias and so on. In order to ensure the robustness of the results of this study, a series of robustness tests are presented below to ensure the robustness of the results.

#### 4.4.1. Parallel trend test.

The event study method is used in this research to generate the interaction terms of the dummy variables of the year and the dummy variables of the treatment group and add them into the model for regression. The interaction coefficient obtained is used to measure the difference between the first treatment group and the control group. The regression model is set as follows:


$$Y_{hct}=\alpha +\sum \beta_{\begin{array}{c} j \end{array}}{Treated}_{c}\cdot {IPost}_{t}+\delta X_{hct}+\tau_{t}+\omega_{h}+\varepsilon_{ct}\;$$

**Fig 1:**
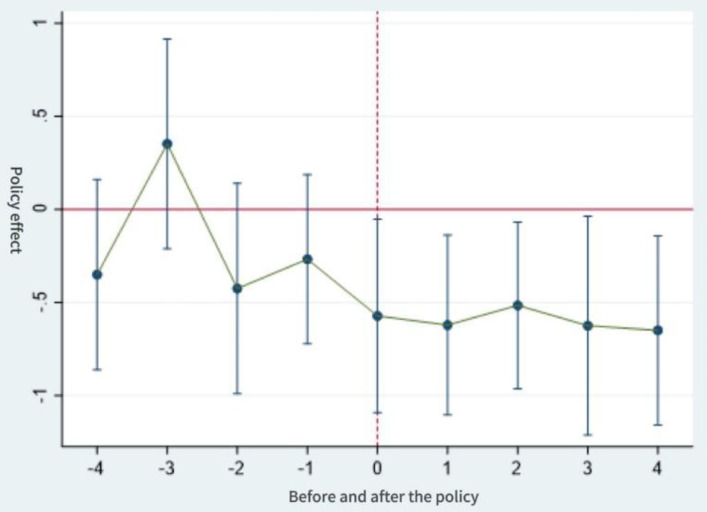
Parallel trend test of economic support.

**Fig 2:**
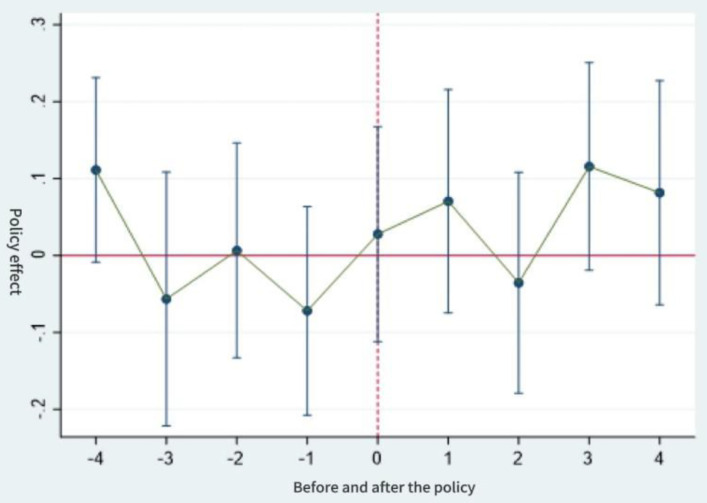
Parallel trend test of emotional support.

**Fig 3:**
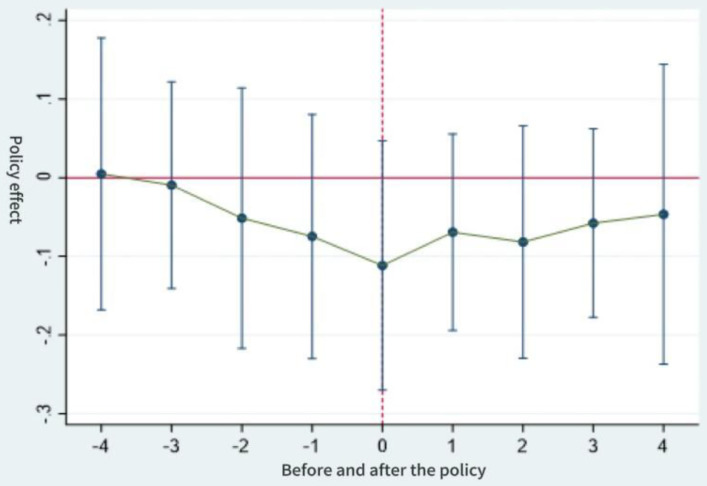
Test for parallel trends in daily care.

Figs 1–3 show the results of the parallel trends test. Prior to the implementation of the long-term care insurance policy, it can be seen that the interaction term coefficient representing the policy effect remained close to zero (with the 95% confidence interval encompassing zero). This indicates that there was no significant difference in intergenerational support behaviors between the control and experimental groups before and after the policy launch, which is consistent with the parallel trends assumption. Furthermore, following the implementation of the long-term care insurance programme in pilot cities, the estimated coefficient for financial support became significant in the same year and in subsequent years, with the zero value no longer falling within the confidence interval. The estimated coefficients for emotional support and daily care also exhibited the characteristic of “non-significance prior to implementation”. All three variables thus satisfy the parallel trends assumption of the difference-in-differences model.

#### 4.4.2. Placebo test.

The counterfactual placebo test was used in this study. The main means was to advance the policy time by four years to observe whether the key variable Did_4 was significant. The following results showed that under the treatment of long-term care insurance implemented four years in advance, the variables of financial support, emotional support and daily care received by the surveyed elderly were not significant, indicating that this study passed the robustness test.

### 4.5. Heterogeneity analysis

Whether the effects of long-term care insurance on intergenerational family support are different due to different geographical and individual characteristics needs to be further explored.

#### 4.5.1. Urban-rural heterogeneity analysis.

There is a dual structure system between urban and rural areas in our country. There are significant differences between urban and rural areas in terms of social economic development level, population age structure, family culture and pension concept. Table 4– shows that long-term care insurance reduces the intergenerational support of the rural elderly by 24.1%, but the impact on the intergenerational support of the elderly is mainly in the urban elderly. In terms of economic support, economic support for the urban elderly was reduced by 34.4% and was stable at the significance level of 5%. In terms of spiritual comfort, the emotional connection between the rural elderly and their children was significantly enhanced, and the frequency of visits increased by 7.4% and was significant at the significance level of 5%. Cultural norms, family structures, and differences in access to social services are key factors that can create disparities between urban and rural areas when long-term care insurance is introduced. Strong family values and close kinship ties in rural areas have solidified the traditional family-based elder care model. This makes adjusting intergenerational relationships complex when introducing long-term care insurance. In contrast, urban areas, influenced by modern culture, have relatively loose family structures, enabling faster acceptance of long-term care insurance. Additionally, urban areas have more developed social service systems than rural regions. These systems include elderly care institutions, community activity centers, and psychological counseling services. They provide seniors with abundant social support resources. Urban seniors have easier access to these services and receive greater emotional care and support. Conversely, the limited availability of social services in rural areas may hinder the effectiveness of long-term care insurance in providing emotional support and fostering intergenerational relationships, leaving rural seniors more dependent on emotional support from their children and family. Heterogeneity analysis for household registration is shown in Table 8.

**Table 7:** Counterfactual check results.

Variable	Economic support	Emotional support	Daily care
Did_4	0.680	0.024	−0.394
	(2.404)	(1.161)	(0.379)
Sequential variable	−0.367	−0.044	0.037
	(0.336)	(0.133)	(0.048)
Constant term	6.456***	1.206*	0.146
	(1.644)	(0.671)	(0.242)
R2	0.077	0.125	0.055

Standard error in parentheses:***p < 0.01,**p < 0.05,*p < 0.1

**Table 8:** Heterogeneity analysis: household registration.

Variable	Agricultural household registration		Urban household registration	
	Economic support	Emotional support	Economic support	Emotional support
Time*treat	−0.241	0.001	−0.364**	0.074**
	(0.176)	(0.040)	(0.164)	(0.036)
Control variable	√	√	√	√
Individual fixation effect	√	√	√	√
Year fixed effect	√	√	√	√
Sample size	15534	21948	3109	5777
R-squared	0.067	0.058	0.039	0.052

Standard error in parentheses:***p < 0.01,**p < 0.05,*p < 0.1

#### 4.5.2. Heterogeneity analysis of health status.

The disability status of elderly parents in each family is different, and the effects of long-term care insurance policies are also different, so it is necessary to conduct a sub-sample regression analysis based on disability. Table 9 shows that long-term care insurance can reduce the intergenerational support of disabled elderly by 3.1% but not significantly, and the impact on the intergenerational support of the elderly is mainly in the urban elderly. In terms of economic support, the economic support for urban elderly people was reduced by 48.4%; In terms of spiritual comfort, the emotional connection between the rural elderly and their children was significantly enhanced, and the frequency of visits increased by 8.4%.

**Table 9:** Heterogeneity analysis: health status.

Variable	Non-disabled		Disabled	
	Economic support	The frequency of visits	Economic support	The frequency of visits
Time*treat	−0.484***	0.084***	−0.031	−0.014
	(0.156)	(0.032)	(0.277)	(0.066)
Control variable	√	√	√	√
Individual fixation effect	√	√	√	√
Year fixed effect	√	√	√	√
Sample size	12473	19138	5990	8587
R-squared	0.074	0.049	0.037	0.059

Standard error in parentheses:***p < 0.01,**p < 0.05,*p < 0.1

### 4.6. Mechanism Inspection

The elderly population who are easy to be troubled by diseases have a great demand for medical services, and medical expenditure accounts for a rising proportion of China’s total medical expenditure. Many scholars have found that long-term care insurance can effectively reduce the elderly’s medical service expenses and the frequency of medical service utilization, and improve the health level of the elderly. Based on this, this paper uses the number of hospitalizations to explore the effect of medical costs on family intergenerational support. According to the results in Table 10, the number of hospitalizations in the pilot area was reduced by 13.1% compared with non-pilot cities, and this result passed the test at the significance level of 5%. The results show that the implementation of long-term care insurance policy has significantly reduced the number of hospitalizations of residents in pilot cities, and the utilization of medical services is one of the channels for the reduction of informal care.

**Table 10:** Mechanism analysis: hospitalization frequency.

Variable	Hospitalizations
Time*treat	−0.131**
	(0.055)
Control variable	√
Individual fixation effect	√
Year fixed effect	√
Sample size	5128
R-squared	0.063

Standard error in parentheses: ***p < 0.01,**p < 0.05,*p < 0.1

## 5. Discussion

This study aims to investigate the impact of long-term care insurance on intergenerational support within families. It explores the factors influencing intergenerational support and the mechanisms at play within the context of accelerated population aging and increasingly nuclear family structures. Additionally, the study assesses whether long-term care insurance alters traditional eldercare models and intergenerational economic and emotional support. By employing a staggered double difference method to quantitatively evaluate the policy effects of long-term care insurance on intergenerational support in pilot cities, we draw the following conclusions:

1) The implementation of long-term care insurance in pilot areas has resulted in a 31% reduction in economic transfers from children to elderly parents, alleviating the financial burden on children.
2) Long-term care insurance has enhanced emotional support between children and parents, increasing the frequency of visits by 5.2%, thereby strengthening intergenerational emotional connections, particularly among urban residents and non-disabled elderly individuals.
3) Long-term care insurance has decreased the duration of care provided by children to their parents, reducing caregiving time by 3.6%, which allows children to focus more on the emotional needs of their parents.
4) The burden-reducing effects of long-term care insurance are particularly pronounced among urban residents, who benefit more in terms of economic support and emotional ties compared to rural elderly populations, highlighting disparities in the allocation of social security resources.

One positive aspect of long-term care insurance is that it breaks the traditional interdependence of “economic support, emotional support, and caregiving” within intergenerational support systems. By sharing financial and caregiving responsibilities, it frees up emotional interactions between generations. This aligns closely with the goals of the “active aging” concept—enhancing the quality of life for older adults and optimizing family support functions. However, caution is warranted, as the policy carries risks of “group differentiation”. In rural areas, where care resources are scarce, some elderly individuals face the dilemma of having policy coverage but inaccessible services, which could exacerbate intergenerational support inequalities between urban and rural areas. On the other hand, elderly individuals with disabilities have a stronger need for “close-contact care”, and while the policy reduces the care time required from their children, it does not address this need. If professional care services fail to meet their personalized needs, some families may find themselves in a situation where professional services are inaccessible and children’s care has decreased. The sense of policy benefit for this group still needs to be enhanced. Based on these findings, the study makes the following recommendations: First, it is imperative to achieve comprehensive coverage of both cash and service benefits as soon as possible. A single welfare payment model cannot meet the diverse needs of the elderly [55], particularly in cities that only provide service benefits, there should be exploration into establishing flexible and diverse benefit schemes to meet the needs of various groups. Furthermore, cross-departmental collaboration should be strengthened to promote resource integration and build a family-centered eldercare service system. Second, the social support system for family caregivers should be improved. The government should provide practical training materials and psychological support to help family caregivers enhance their caregiving skills. A subsidy system for informal caregivers who provide uncompensated care to family members should be established to alleviate their financial burden. Finally, there should be a continuous optimization of both informal and formal caregiving services, with an emphasis on developing community-based eldercare systems and enhancing the accessibility of professional nursing services. The applicability of long-term care insurance should be broadened, particularly in rural areas, to better meet the caregiving needs of diverse populations. These measures will contribute to enhancing the quality and effectiveness of intergenerational support within families, thereby mitigating the challenges posed by an aging population.

## 6. Limitations and future directions

This study relies on the CHARLS database from 2011 to 2020, which encompasses only a subset of cities piloting long-term care insurance, lacking data from cities such as Shihezi, Nantong, and Changchun. This limitation results in a lack of comprehensiveness in the study sample, making it impossible to evaluate the policy effects of long-term care insurance on intergenerational support for the elderly in these cities. Additionally, research on upward intergenerational caregiving predominantly measures the caregiving time provided by children to elderly parents. However, the CHARLS database does not differentiate between the caregiving time provided by children and that provided by grandchildren, hindering an accurate representation of the specifics of intergenerational support. Finally, this study focuses solely on the upward support provided by children to the elderly and does not consider the downward support that elderly individuals may provide to their children and grandchildren, thus limiting a comprehensive understanding of the intergenerational support relationships within families.

Future research should focus on four key areas: First, when analyzing intergenerational support, it is essential to integrate social influences with individual factors, coupled with macro-level policy analysis to gain deeper insights into micro-level individual behaviors. Second, studies should evaluate the impact and effectiveness of mediating variables such as economic support, emotional support, and caregiving time on the life satisfaction of children. As the health status of elderly individuals’ changes, the manner and extent of support provided by children may also evolve, influencing the dynamic characteristics of intergenerational support. Third, future research should explore other potential factors to gain a more comprehensive understanding of the complex relationship between intergenerational support and children’s life satisfaction. Lastly, attention to the design and optimization of social support policies aimed at meeting the needs of the elderly will contribute to enhancing overall social welfare.
